# Oxidative Cyclization of 5*H*-Chromeno[2,3-*b*]pyridines to Benzo[*b*]chromeno[4,3,2-*de*][1,6]naphthyridines, Their NMR Study and Computer Evaluation as Material for LED

**DOI:** 10.3390/molecules27134156

**Published:** 2022-06-28

**Authors:** Yuliya E. Ryzhkova, Fedor V. Ryzhkov, Artem N. Fakhrutdinov, Michail N. Elinson

**Affiliations:** N. D. Zelinsky Institute of Organic Chemistry, Russian Academy of Sciences, 47 Leninsky Prospekt, 119991 Moscow, Russia; yu_ryzhkova@ioc.ac.ru (Y.E.R.); ryzhkovfv@ioc.ac.ru (F.V.R.); fak.part@gmail.com (A.N.F.)

**Keywords:** benzo[*b*]chromeno[4,3,2-*de*][1,6]naphthyridine, chromeno[2,3-*b*]pyridine, computer evaluation, formic acid, NMR study, oxidative cyclization

## Abstract

Oxidative cyclization is one of the most significant reactions in organic synthesis. Naphthyridine derivatives are often used as luminescence materials in molecular recognition because of their rigid planar structure and as new drugs. Organic light-emitting diodes (OLEDs) have rapidly grown as one of the leading technologies for full-color display panels and eco-friendly lighting sources. In this work, we propose the synthesis of previously unknown benzo[*b*]chromeno[4,3,2-*de*][1,6]naphthyridines via intermolecular oxidative cyclization of 5-(2-hydroxy-6-oxocyclohexyl)-5*H*-chromeno[2,3-*b*]pyridines in formic acid. The investigation of the reaction mechanism using ^1^H-NMR monitoring made it possible to confirm the proposed mechanism of the transformation. The structure of synthesized benzo[*b*]chromeno[4,3,2-*de*][1,6]naphthyridines was confirmed by 2D-NMR spectroscopy. Such a rigid geometry of synthesized compounds is desired to minimize non-radiative energy losses in OLEDs. The quantum chemical calculations are also presented in the study.

## 1. Introduction

Oxidative cyclization is one of the most significant reactions in organic synthesis [1]. These transformations are often facilitated by expensive or toxic catalytic systems, such as palladium salts, lead acetates, and other transition-metal catalysts [2,3]. The advances in photocatalytic oxidative cyclizations catalyzed by titanium dioxide are also known [4]. Oxidative cyclizations occur widely during natural product biosynthesis [5,6] or biomimetic synthesis [7,8]. Thus, oxidative cyclization is one of the most common methods for synthesizing different ring systems.

Fused (condensed) heterocycles are systems constructed by combining two or more rings [9]. Due to their unique structural features, highly condensed polycyclic compounds are increasingly crucial in polymer chemistry, materials science, and pharmaceutical chemistry. Naphthyridines are nitrogen-containing heterocyclic analogs of naphthalenes [10]. They contain a nitrogen atom in each of their benzene rings. Naphthyridine derivatives are often used as luminescence materials because of their rigid planar structure [11] and as a scaffold for new drugs in medicinal chemistry [12].

Organic light-emitting diodes (OLEDs) have rapidly grown as one of the leading technologies for full-color display panels and eco-friendly lighting sources due to their outstanding features, including superior color quality, wide viewing angle, mercury-free manufacture, fascinating flexibility, etc. [13]. Various materials, device architectures, and processing techniques have been investigated for optimizing device performance to fulfill the requirements of lighting and display applications.

Fluorescent organic molecules can change their fluorescence properties, such as absorption, excitation, and emission [14]. The planar π-conjugated and polycyclic aromatic molecules are widely used in luminescent material. Because they would be strong absorption and emission wavelength with high-intensity concentration and higher luminescence quantum yield, but luminescence is quenched in solid-state or high-intensity media.

Finally, synthesizing new polycondensed naphthyridine derivatives is a modern relevant goal for organic chemistry.

## 2. Results and Discussion

### 2.1. Intramolecular Oxidative Cyclization of 5H-Chromeno[2,3-b]pyridines to Benzo[b]chromeno[4,3,2-de][1,6]naphthyridines

**Scheme 1:**
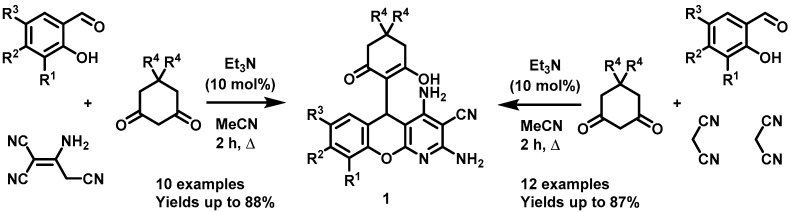
Two methods of the (PASE) synthesis of 5*H*-chromeno[2,3-*b*]pyridine-3-carbonitriles **1**.

Previously we reported two methods of the pot, atom, and step economy (PASE) synthesis of 5-(2-hydroxy-6-oxocyclohexyl)-5*H*-chromeno[2,3-*b*]pyridine-3-carbonitriles **1** (Scheme 1) [15,16,17].

The synthesized chromeno[2,3-*b*]pyridines **1** have in their structure a hydroxyl group and an amino group located at a distance that does not contradict the intramolecular interaction. However, this cyclization did not occur during multicomponent transformations. In this regard, we decided to study the possible cyclization of already-synthesized compounds **1** and find optimal conditions for the synthesis.

Initially, to examine the intramolecular cyclization of 5-(2-hydroxy-6-oxocyclohexyl)-5*H*-chromeno[2,3-*b*]pyridine-3-carbonitriles **1**, we chose 2,4-diamino-5-(2-hydroxy-4,4-dimethyl-6-oxocyclohex-1-en-1-yl)-5*H*-chromeno[2,3-*b*]pyridine-3-carbonitrile **1a** as an object to find the cyclization conditions (Table 1).

**Scheme 2:**
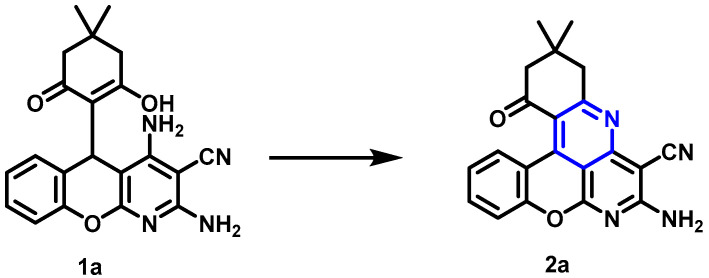
Intramolecular oxidative cyclization of 5*H*-chromeno[2,3-*b*]pyridine **1a**.

First, several experiments were carried out with dehydration agents (Scheme 2, Table 1, Entries 1–3). In these reactions, compound **1a** was isolated unchanged. TsOH, HCl, and P_2_O_5_ did not lead to interaction of hydroxyl and amino groups. Further, the process was carried out in formic acid and formamide (Scheme 2, Table 1, Entries 4–9). In these solvents, the cyclization took place. More than that, in both solvents the further oxidation was recorded.

It is supposed that strong polar media is required for the reaction. Possibly, formic acid and formamide interact with hydroxyl and amino groups with formation of the cyclic intermediate. The aromaticity of benzo[*b*]chromeno[4,3,2-*de*][1,6]naphthyridine **2a** promotes further oxidation by dissolved oxygen.

Heating of the chromeno[2,3-*b*]pyridine **1a** in formamide at 100 °C showed good yields (31–58%) of benzo[*b*]chromeno[4,3,2-*de*][1,6]naphthyridine **2a**, and the reaction reflux resulted in partial decomposition of chromeno[2,3-*b*]pyridine **1a** (Scheme 2, Table 1, Entries 5–7). Carrying out the reaction in formic acid made it possible to obtain better yields of **2a** (78–81%). Apparently, protic polar media is more favourable for the cyclization than aprotic one. An increase in the heating time to 4 and 6 h led to the appearance of side processes and unwanted signals in the proton NMR spectra (Scheme 2, Table 1, Entries 4, 8 and 9).

Thus, the best result in the synthesis of benzo[*b*]chromeno[4,3,2-*de*][1,6]naphthyridine **2a** was achieved by refluxing chromeno[2,3-*b*]pyridine **1a** in formic acid for 2 h (Scheme 2, Table 1, Entry 4). The yield of compound **2a** was 78%.

Benzo[*b*]chromeno[4,3,2-*de*][1,6]naphthyridines **2a**–**f** were synthesized under optimal conditions from 5-(2-hydroxy-6-oxocyclohexyl)-5*H*-chromeno[2,3-*b*]pyridines **1a**–**f** in 61–85% yields (Table 2). It is seemed that electron-donating group in R^1^ position slightly decreases the yields while donating group in R^2^ position increases them. This fact may be explained by occurrence of mesomeric effect on amino group during cyclization.

The structure of the obtained compounds **2a**–**f** was confirmed by ^1^H and ^13^C NMR data, IR spectroscopy, mass spectrometry, and elemental analysis. In addition, the structure study for compound **2a** was also carried out using two-dimensional NMR spectroscopy techniques (see Section 2.2).

The intramolecular oxidative cyclization involves the use of simple equipment and is easy to implement; the final compounds are isolated by adding a small amount of a mixture of ethanol-water (1:1 vol.) to the reaction mass and do not require chromatographic purification or recrystallization.

### 2.2. 2D NMR Study of the Structure of Compound **2a**

The structure of compound **2a** was confirmed by hydrogen and carbon NMR spectroscopy.

**Figure 1:**
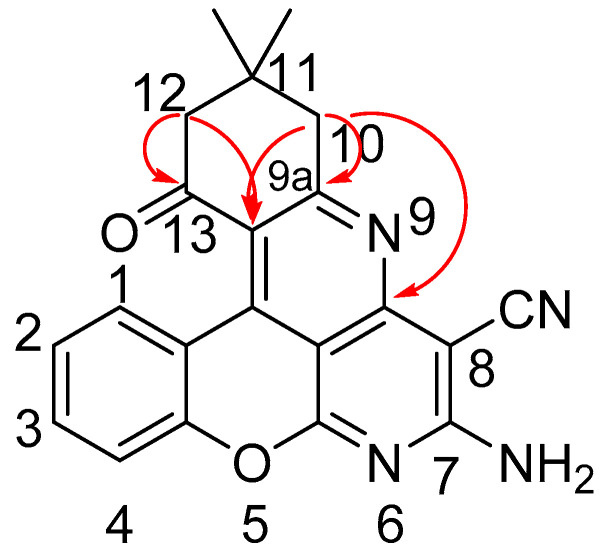
The structure of compound **2a**. Key ^1^H-^13^C-HMBC spectrum correlations established by NMR are shown by arrows.

The proton spectrum showed four multiplets from aromatic hydrogens and three singlets from aliphatic fragments. All 20 signals from the compound were found in the carbon NMR spectrum. The signal assignment was carried out on the base of 2D NMR spectra (Figure 1). In the ^1^H-^13^C HMBC spectrum, the coupling between H^10^ of cyclohexenone and C^8a^ of pyridine ring was observed at δ_H_/δ_C_ 3.09/155.8 ppm, that proves the formation of another cycle by bonding of nitrogen (9) and carbon (9a).

Complete correlation of signals in ^1^H and ^13^C-NMR spectra of benzo[*b*]chromeno[4,3,2-*de*][1,6]naphthyridine **2a**:

^1^H-NMR (600 MHz, DMSO-*d*_6_) δ: 8.03 (dd, ^3^*J* = 8.3 Hz, ^4^*J* = 1.5 Hz, 1H, H^1^), 7.82 (s, 2H, NH_2_), 7.70 (ddd, ^3^*J* = 8.5 Hz, ^3^*J* = 7.1 Hz, ^4^*J* = 1.5 Hz, 1H, H^3^), 7.53 (dd, ^3^*J* = 8.3 Hz, ^4^*J* = 1.2 Hz, 1H, H^4^), 7.31 (ddd, ^3^*J* = 8.4 Hz, ^3^*J* = 7.2 Hz, ^4^*J* = 1.3 Hz, 1H, H^2^), 3.09 (s, 2H, H^10^), 2.76 (s, 2H, H^12^), 1.10 (s, 6H, CH_3_) ppm.

^13^C-NMR (151 MHz, DMSO-*d*_6_) δ: 198.1 (C^13^), 169.3 (C^9a^), 161.9, 159.1 (C^5a^, C^7^), 155.8 (C^8a^), 152.2 (C^4a^), 139.5 (C^13b^), 133.9 (C^3^), 129.9 (C^1^), 123.8 (C^2^), 117.8 (C^4^), 117.6 (C^13a^), 116.04 (C^1a^), 115.99 (CN), 104.0 (C^5b^), 76.3 (C^8^), 53.6 (C^12^), 48.1 (C^10^), 32.3 (C^11^), 27.9 (CH_3_) ppm.

Thus, the structure of compound **2a** is unambiguously confirmed.

One- and two-dimensional (2D) NMR spectra of compound **2a** are presented in Supplementary Materials (Figures S13–S17).

### 2.3. ^1^H NMR Reaction Monitoring and Mechanism of the Process

**Figure 2:**
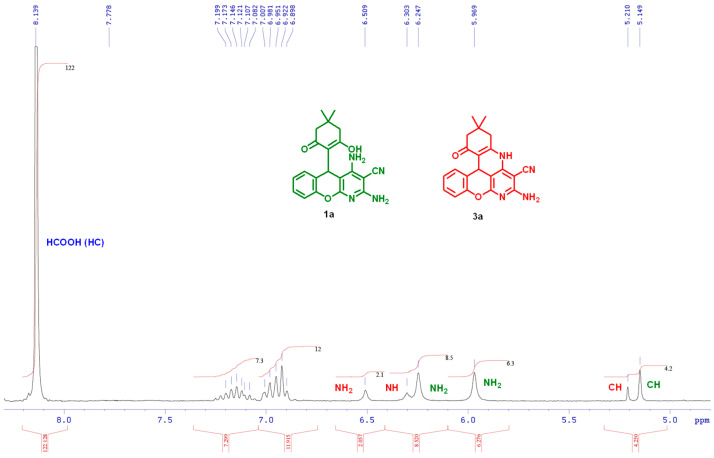
Representative ^1^H-NMR spectrum of intramolecular oxidative cyclization in DMSO-*d_6_* at 353 K recorded 60 min after dissolution.

To study the mechanism of the process, we carried out real-time ^1^H NMR monitoring of the reaction. For this, the intramolecular oxidative cyclization was carried out in a NMR tube in DMSO-*d_6_* by adding of 26-fold molar excess of formic acid relative to compound **1a** (Figure 2).

To reduce the influence of sample preparation, the transformation of chromeno[2,3-*b*]pyridine **1a** was carried out and monitored directly in an NMR sample tube into a spectrometer in DMSO-*d_6_* at 80 °C to slow down the reaction.

The NMR study recorded three significant components: 5*H*-chromeno[2,3-*b*]pyridine **1a**, formic acid, and intermediate **3**. A representative ^1^H-NMR spectrum of oxidative cyclization with the assignment of peaks showed in Figure 2. ^1^H-NMR spectra of the monitoring are presented in Supplementary Materials (Figures S18 and S19).

As shown in Figure 2, formic acid concentration does not change compound **1a** is consumed slowly, and intermediate cyclized compound **3a** is formed. Based on these data, we can conclude that cyclization occurs first, and then oxidation.

**Scheme 3:**
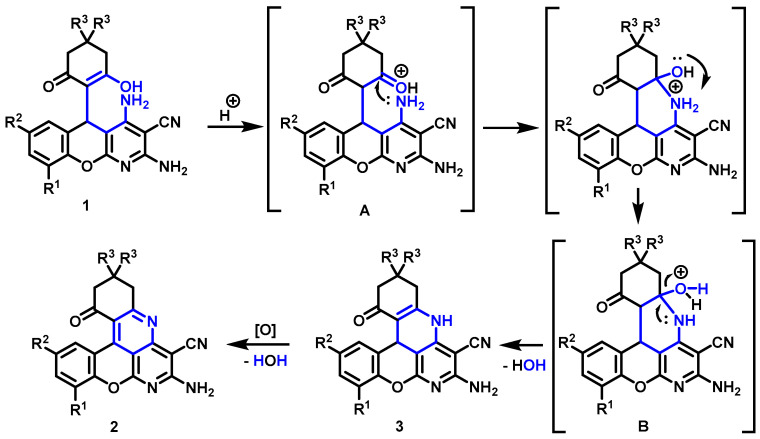
Intramolecular oxidative cyclization of 5*H*-chromeno[2,3-*b*]pyridine **1a** in formic acid.

Based on the above data, the following mechanism of intramolecular oxidative cyclization of 5-(2-hydroxy-6-oxocyclohexyl)-5*H*-chromeno[2,3-*b*]pyridines **1** to 10,11,12,13-tetrahydrobenzo[*b*]chromeno[4,3,2-*de*][1,6]naphthyridines **2** (Scheme 3).

In the first stage, the keto group of the 1,3-cyclohexanedione fragment of compound **1** is protonated. Further, cation **A** undergoes cyclization with the formation of a new tetrahydropyridine ring and tautomerization with the appearance of a good leaving group (intermediate **B**). Cleavage of a water molecule leads to 9,10,11,12,13,13*b*-hexahydrobenzo[*b*]chromeno[4,3,2-*de*][1,6]naphthyridine 3, which is oxidized by atmospheric oxygen to 10,11,12,13-tetrahydrobenzo[*b*]chromeno[4,3,2-*de*][1,6]naphthyridine **2**.

### 2.4. Computer Evaluation as Material for LED

There are only a few examples of synthesizing similar 1,6-naphthyridines in the literature [12,18,19,20,21]. Often such compounds are investigated as materials for LEDs or evaluated for their fluorescent properties.

During experiments, we noticed that the solute of the synthesized compound had optical effects. It turned out that a plethora of condensed aromatic compounds can display fluorescence. More than that, such a rigid geometry is desired to minimize non-radiative energy losses in organic light-emitting devices (OLEDs). Thus, azatetracenes are effective OLED materials with high current efficiency [22]. Rubrene [23] and chromenes [24,25] are known as red OLEDs.

The easiest way to estimate the suitability of an organic compound to be applied as a light-emitting material is to calculate the Highest Occupied Molecular Orbital (HOMO) and the Lowest Unoccupied Molecular Orbital (LUMO). It is important for an organic–organic interface. HOMO and LUMO difference connected to the rate of nonradiative decay. It increases with decreasing energy difference.

The quantum chemical calculations of HOMO and LUMO of synthesized compounds were performed. The results of the calculations are presented in Table 3, and frontier orbitals of several studied compounds are shown in Table 4. The results show that energy gap (ΔE(L − H)) is broad in comparison to Rubrene, but it is closer to 4,4′-bis(N-phenyl-1-naphthylamino)biphenyl (NPB) [26]. In case of OLED, it may be interesting to further investigate the synthesized compounds in multi-layer materials.

## 3. Materials and Methods

### 3.1. General Information

The solvents and reagents were purchased from commercial sources and used as received. 5-(2-Hydroxy-6-oxocyclohexyl)-5*H*-chromeno[2,3-*b*]pyridine-3-carbonitriles **1** were obtained according to the literature [8,9].

All melting points were measured with Gallenkamp melting-point apparatus (Gallenkamp and Co., Ltd., London, UK) and were uncorrected. ^1^H and ^13^C NMR spectra were recorded in DMSO-*d_6_* with Bruker AM300 and Bruker AV500 spectrometers (Bruker Corporation, Billerica, MA, USA) at ambient temperature. Chemical shift values are relative to Me_4_Si. The numbering of atoms of compounds **2a**–**f**, used in the interpretation of ^13^C-NMR spectra, is shown in Figure 1. Two-dimensional (2D) NMR spectra were registered with a Bruker AV600 spectrometer (Bruker Corporation, Billerica, MA, USA). ^1^H NMR monitoring spectra were registered with a Bruker AM300 spectrometer (Bruker Corporation, Billerica, MA, USA). The IR spectrum was recorded with a Bruker ALPHA-T FT-IR spectrometer (Bruker Corporation, Billerica, MA, USA) in KBr pellet. MS spectra (EI = 70 eV) were obtained directly with a Kratos MS-30 spectrometer (Kratos Analytical Ltd., Manchester, UK). For elemental analysis, a 2400 Elemental Analyzer (Perkin Elmer Inc., Waltham, MA, USA) was used.

NWChem software was used for the calculation of the orbitals [27]. The structures of **2a**–**f** were optimized and the required energies were calculated using the Hartree–Fock theory method with the 6-311G(d) basic set.

### 3.2. Intramolecular Oxidative Cyclization of 5-(2-Hydroxy-6-Oxocyclohexyl)-5H-Chromeno[2,3-b]pyridine-3-Carbonitrile **1**

A solution of 5-(2-hydroxy-6-oxocyclohexyl)-5*H*-chromeno[2,3-*b*]pyridine-3-carbonitrile **1** (0.5 mmol) was refluxed in formic acid (2.5 mL) for 2 h. After the completion of the reaction, a mixture of ethanol-water (1:1 vol., 2 mL), the formed precipitate of benzo[*b*]chromeno[4,3,2-*de*][1,6]naphthyridine **2** was separated by filtration, washed with cold ethanol (3 mL) and dried.

**7-Amino-11,11-dimethyl-13-oxo-10,11,12,13-tetrahydrobenzo[*b*]chromeno[4,3,2-*de*][1,6]naphthyridine-8-carbonitrile 2a**, (yellow powder, 0.139 g, 78%), mp > 350 °C (from formic acid-ethanol-H_2_O), FTIR (KBr) cm^−1^: 3408, 3220, 2212, 1631, 1596, 1579, 1540, 1478. ^1^H-NMR (300 MHz, DMSO-*d_6_*) *δ* 1.10 (s, 6H, 2 CH_3_), 2.75 (s, 2H, CH_2_), 3.08 (s, 2H, CH_2_), 7.30 (t, ^3^*J* = 8.2 Hz, 1H, CH Ar), 7.50 (d, ^3^*J* = 8.2 Hz, 1H, CH Ar), 7.69 (t, ^3^*J* = 8.2 Hz, 1H, CH Ar), 7.81 (br s, 2H, NH_2_), 8.01 (d, ^3^*J* = 8.2 Hz, 1H, CH Ar) ppm. ^13^C-NMR (126 MHz, DMSO-*d_6_*) *δ* 27.9 (2C, 2 CH_3_), 32.4 (C(11)-(CH_3_)_2_), 48.0 (C(10)H_2_), 53.6 (C(12)H_2_), 76.2 (C(8)-CN), 104.0 (C(5^b^)), 116.0 (2C, CN and C(1^a^)), 117.6 (C(13^a^)), 117.8 (C(4)H), 123.8 (C(2)H), 129.9 (C(1)H), 133.9 (C(3)H), 139.4 (C(13^b^)), 152.2 (C(4^a^)), 155.8 (C(8^a^)-N), 159.1 (C(7)-NH_2_), 161.9 (C(5^a^)), 169.3 (C(9^a^)), 198.1 (C(13)=O) ppm. MS (EI, 70 eV) *m*/*z* (%): 356 ([M]^+^, 100), 328 (31), 300 (67), 271 (29), 237 (4), 189 (3), 164 (2), 126 (1), 56 (5), 41 (11). Anal. calcd. for C_21_H_16_N_4_O_2_: C, 70.77; H, 4.53; N, 15.72%; found: C, 70.71; H, 4.58; N, 15.70%.

**7-Amino-4-methoxy-11,11-dimethyl-13-oxo-10,11,12,13-tetrahydrobenzo[*b*]chromeno[4,3,2-*de*][1,6]naphthyridine-8-carbonitrile 2b**, (yellow powder, 0.126 g, 65%), mp > 350 °C (from formic acid-ethanol-H_2_O), FTIR (KBr) cm^−1^: 3344, 2956, 2211, 1620, 1598, 1586, 1544, 1273. ^1^H-NMR (300 MHz, DMSO-*d_6_*) *δ* 1.10 (s, 6H, 2 CH_3_), 2.75 (s, 2H, CH_2_), 3.08 (s, 2H, CH_2_), 3.95 (s, 3H, OCH_3_), 7.21 (t, ^3^*J* = 8.2 Hz, 1H, CH Ar), 7.35 (d, ^3^*J* = 8.2 Hz, 1H, CH Ar), 7.50 (d, ^3^*J* = 8.2 Hz, 1H, CH Ar), 7.81 (br s, 2H, NH_2_) ppm. ^13^C-NMR (75 MHz, DMSO-*d_6_*) *δ* 28.0 (2C, 2 CH_3_), 32.3 (C(11)-(CH_3_)_2_), 48.1 (C(10)H_2_), 53.6 (C(12)H_2_), 56.4 (OCH_3_), 76.3 (C(8)-CN), 103.9 (C(5^b^)), 115.5 (C(3)H), 116.0 (CN), 116.7 (C(1^a^)), 117.9 (C(13^a^)), 120.7 (C(1)), 123.4 (C(2)H), 139.6 (C(13^b^)), 142.2 (C(4)-OCH_3_), 147.9 (C(4^a^)), 155.8 (C(8^a^)-N), 158.8 (C(7)-NH_2_), 161.9 (C(5^a^)), 169.3 (C(9^a^)), 198.1 (C(13)=O) ppm. MS (EI, 70 eV) *m*/*z* (%): 386 ([M]^+^, 100), 358 (25), 330 (58), 304 (13), 267 (18), 204 (6), 177 (4), 164 (4), 83 (7), 41 (9). Anal. calcd. for C_22_H_18_N_4_O_3_: C, 68.38; H, 4.70; N, 14.50%; found: C, 68.32; H, 4.74; N, 14.47%.

**7-Amino-2-bromo-11,11-dimethyl-13-oxo-10,11,12,13-tetrahydrobenzo[*b*]chromeno[4,3,2-*de*][1,6]naphthyridine-8-carbonitrile 2c**, (yellow powder, 0.185 g, 85%), mp > 350 °C (from formic acid-ethanol-H_2_O), FTIR (KBr) cm^−1^: 3365, 3177, 2218, 1647, 1611, 1590, 1569, 1396. ^1^H-NMR (300 MHz, DMSO-*d_6_*) *δ* 1.10 (s, 6H, 2 CH_3_), 2.78 (s, 2H, CH_2_), 3.09 (s, 2H, CH_2_), 7.50 (d, ^3^*J* = 8.8 Hz, 1H, CH Ar), 7.79–7.85 (m, 3H, NH_2_ + CH Ar), 8.21 (d, ^4^*J* = 1.5 Hz, 1H, CH Ar) ppm. ^13^C-NMR (75 MHz, DMSO-*d_6_*) *δ* 27.9 (2C, 2 CH_3_), 32.3 (C(11)-(CH_3_)_2_), 48.2 (C(10)H_2_), 53.7 (C(12)H_2_), 76.4 (C(8)-CN), 104.1 (C(5^b^)), 115.5 (C(1^a^)), 115.9 (CN), 117.7 (C(13^a^)), 118.0 (C(2)-Br), 120.1 (C(4)H), 132.0 (C(1)H), 136.2 (C(3)H), 138.3 (C(13^b^)), 151.5 (C(4^a^)), 155.8 (C(8^a^)-N), 158.9 (C(7)-NH_2_), 161.8 (C(5^a^)), 169.5 (C(9^a^)), 198.3 (C(13)=O) ppm. MS (EI, 70 eV) *m*/*z* (%): 436 (^81^Br, [M]^+^, 100), 434 (^79^Br, [M]^+^, 96), 380 (^81^Br, 81), 378 (^79^Br, 82), 354 (^81^Br, 18), 352 (^79^Br, 16), 328 (25), 317 (^81^Br, 13), 315 (^79^Br, 12), 271 (14), 201 (4), 170 (16), 128 (3), 39 (14). Anal. calcd. for C_21_H_15_BrN_4_O_2_: C, 57.95; H, 3.47; N, 12.87%; found: C, 57.89; H, 3.51; N, 12.85%.

**7-Amino-2,4-dibromo-11,11-dimethyl-13-oxo-10,11,12,13-tetrahydrobenzo[*b*]chromeno-[4,3,2-*de*][1,6]naphthyridine-8-carbonitrile 2d**, (yellow powder, 0.183 g, 71%), mp > 350 °C (from formic acid-ethanol-H_2_O), FTIR (KBr) cm^−1^: 3398, 3226, 2210, 1638, 1610, 1560, 1393, 1249. ^1^H-NMR (300 MHz, DMSO-*d_6_*) *δ* 1.09 (s, 6H, 2 CH_3_), 2.79 (s, 2H, CH_2_), 3.10 (s, 2H, CH_2_), 8.01 (br s, 2H, NH_2_), 8.21 (d, ^4^*J* = 2.2 Hz, 1H, CH Ar), 8.25 (d, ^4^*J* = 2.2 Hz, 1H, CH Ar) ppm. ^13^C-NMR (75 MHz, DMSO-*d_6_*) *δ* 27.9 (2C, 2 CH_3_), 32.3 (C(11)-(CH_3_)_2_), 48.2 (C(10)H_2_), 53.7 (C(12)H_2_), 76.6 (C(8)-CN), 104.3 (C(5^b^)), 111.9 (C(4)-Br), 115.4 (C(1^a^)), 115.7 (CN), 118.0 (C(2)-Br), 119.3 (C(13^a^)), 131.7 (C(1)H), 138.0 (C(13^b^)), 138.3 (C(3)H), 148.3 (C(4^a^)), 155.7 (C(8^a^)-N), 158.6 (C(7)-NH_2_), 161.9 (C(5^a^)), 169.6 (C(9^a^)), 198.2 (C(13)=O) ppm. MS (EI, 70 eV) *m*/*z* (%): 516 (^81^Br, ^81^Br, [M]^+^, 5), 514 (^81^Br, ^79^Br, [M]^+^, 11), 512 (^79^Br, ^79^Br, [M]^+^, 6), 460 (^81^Br, ^81^Br, 2), 458 (^81^Br, ^79^Br, 5), 456 (^79^Br, ^79^Br, 3), 397 (^81^Br, ^81^Br, 5), 395 (^81^Br, ^79^Br, 11), 393 (^79^Br, ^79^Br, 6), 371 (^81^Br, ^81^Br, 2), 369 (^81^Br, ^79^Br, 5), 367 (^79^Br, ^79^Br, 3), 314 (3), 235 (2), 208 (3), 158 (100), 130 (45), 41 (24). Anal. calcd. for C_21_H_14_Br_2_N_4_O_2_: C, 49.06; H, 2.74; N, 10.90%; found: C, 49.00; H, 2.78; N, 10.87%.

**7-Amino-2-bromo-4-methoxy-11,11-dimethyl-13-oxo-10,11,12,13-tetrahydrobenzo[b]chromeno[4,3,2-*de*][1,6]naphthyridine-8-carbonitrile 2e**, (yellow powder, 0.142 g, 61%), mp > 350 °C (from formic acid-ethanol-H_2_O), FTIR (KBr) cm^−1^: 3355, 2956, 2211, 1638, 1614, 1582, 1543, 1218. ^1^H-NMR (300 MHz, DMSO-*d_6_*) *δ* 1.09 (s, 6H, 2 CH_3_), 2.77 (s, 2H, CH_2_), 3.08 (s, 2H, CH_2_), 3.97 (s, 3H, OCH_3_), 7.52 (s, 1H, CH Ar), 7.66 (s, 1H, CH Ar), 7.87 (br s, 2H, NH_2_) ppm. ^13^C-NMR (75 MHz, DMSO-*d_6_*) *δ* 27.9 (2C, 2 CH_3_), 32.3 (C(11)-(CH_3_)_2_), 48.2 (C(10)H_2_), 53.7 (C(12)H_2_), 56.8 (OCH_3_), 76.4 (C(8)-CN), 104.1 (C(5^b^)), 115.2 (C(1^a^)), 115.9 (CN), 117.8 (C(13^a^)), 118.1 (2C, C(1)H and C(2)-Br), 122.7 (C(3)H), 138.4 (C(13^b^)), 141.7 (C(4)-OCH_3_), 148.8 (C(4^a^)), 155.8 (C(8^a^)-N), 158.5 (C(7)-NH_2_), 161.8 (C(5^a^)), 169.5 (C(9^a^)), 198.2 (C(13)=O) ppm. MS (EI, 70 eV) *m*/*z* (%): 466 (^81^Br, [M]^+^, 100), 464 (^79^Br, [M]^+^, 98), 410 (^81^Br, 16), 408 (^79^Br, 14), 355 (15), 330 (13), 257 (14), 222 (19), 178 (14), 153 (11), 90 (11), 41 (90). Anal. calcd. for C_22_H_17_BrN_4_O_3_: C, 56.79; H, 3.68; N, 12.04%; found: C, 56.72; H, 3.73; N, 12.02%.

**7-Amino-13-oxo-10,11,12,13-tetrahydrobenzo[*b*]chromeno[4,3,2-*de*][1,6]naphthyridine-8-carbonitrile 2f**, (yellow powder, 0.130 g, 79%), mp > 350 °C (from formic acid-ethanol-H_2_O), FTIR (KBr) cm^−1^: 3385, 3208, 2214, 1638, 1598, 1579, 1542, 1479. ^1^H-NMR (300 MHz, DMSO-*d_6_*) *δ* 2.06–2.20 (m, 2H, CH_2_), 2.81 (t, ^3^*J* = 6.6 Hz, 2H, CH_2_), 3.07 (t, ^3^*J* = 6.6 Hz, 2H, CH_2_), 7.30 (t, ^3^*J* = 8.1 Hz, 1H, CH Ar), 7.52 (d, ^3^*J* = 8.1 Hz, 1H, CH Ar), 7.70 (t, ^3^*J* = 8.1 Hz, 1H, CH Ar), 7.81 (br s, 2H, NH_2_), 8.05 (d, ^3^*J* = 8.1 Hz, 1H, CH Ar) ppm. ^13^C-NMR (75 MHz, DMSO-*d_6_*) *δ* 20.2 (C(11)H_2_), 34.5 (C(10)H_2_), 39.5 (C(12)H_2_), 76.2 (C(8)-CN), 104.1 (C(5^b^)), 116.0 (2C, CN and C(1^a^)), 117.8 (C(4)H), 118.5 (C(13^a^)), 123.9 (C(2)H), 129.9 (C(1)H), 133.9 (C(3)H), 139.8 (C(13^b^)), 152.2 (C(4^a^)), 155.6 (C(8^a^)-N), 159.1 (C(7)-NH_2_), 161.9 (C(5^a^)), 170.9 (C(9^a^)), 198.2 (C(13)=O) ppm. MS (EI, 70 eV) *m*/*z* (%): 328 ([M]^+^, 78), 300 (100), 271 (30), 264 (8), 237 (6), 216 (5), 189 (10), 164 (10), 136 (5), 28 (26). Anal. calcd. for C_19_H_12_N_4_O_2_: C, 69.51; H, 3.68; N, 17.06%; found: C, 69.46; H, 3.72; N, 17.03%.

## 4. Conclusions

In summary, the intramolecular oxidative cyclization of 5-(2-hydroxy-6-oxocyclohexyl)-5*H*-chromeno[2,3-*b*]pyridine-3-carbonitriles into previously unknown benzo[*b*]chromeno[4,3,2-*de*][1,6]naphthyridines has been found. The developed approach is facile and easy for isolating final compounds directly from the reaction mixture using water–ethanol mixture addition, and the yields of final compounds are 61–85%.

Two-dimensional (2D) NMR spectroscopy unambiguously confirmed the proposed structure of synthesized benzo[*b*]chromeno[4,3,2-*de*][1,6]naphthyridines.

During the investigation of the reaction mechanism using ^1^H-NMR monitoring at heating, it was determined that the reaction occurs in polar solvents. The protic solvent is more preferable than aprotic one. During the intramolecular process, the starting chromeno[2,3-*b*]pyridine is first cyclized, and then the cyclic intermediate is oxidized.

Such a rigid geometry of synthesized compounds may be desired in organic light-emitting devices (OLEDs). To evaluate applicability of the compounds the quantum chemical calculations were performed. The results show broad energy gap. In case of OLEDs, it may be interesting to further investigate the synthesized compounds in multi-layer materials.

## Figures and Tables

**Table 1:** Optimization of intramolecular oxidative cyclization conditions ^1^.

Entry	Solvent	Catalyst	Time (h)	Temp. (°C)	Yield (%)
1	EtOH	TsOH × H_2_O	2	78	–
2	EtOH	HCl	2	78	–
3	EtOH	P_2_O_5_	2	78	–
4	HCOOH	–	2	101	78 ^2^
5	HCONH_2_	–	2	100	54 ^2^
6	HCONH_2_	–	4	100	58 ^2^
7	HCONH_2_	–	2	210	31
8	HCOOH	–	4	101	79 ^2^
9	HCOOH	–	6	101	81

^1^ Reaction conditions: 5*H*-chromeno[2,3-*b*]pyridine **1a** (0.5 mmol), catalyst (10 mol%) or without it and solvent (2.5 mL) were heated. ^2^ Isolated yield, in other cases NMR data.

**Table 2:** Intramolecular oxidative cyclization of 5*H*-chromeno[2,3-*b*]pyridines **1a**–**f** ^1^.

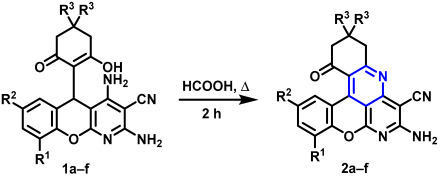
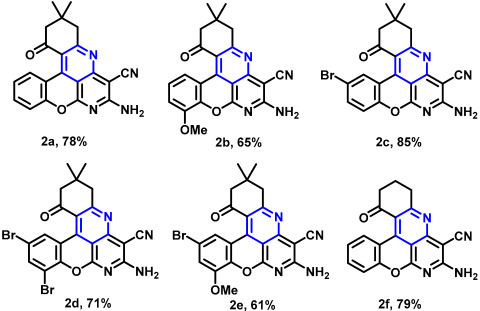

^1^ Reaction conditions: 5*H*-chromeno[2,3-*b*]pyridine **1a**–**f** (0.5 mmol) and formic acid (2.5 mL) were refluxed for 2 h, then 2 mL of of ethanol–water mixture (1:1 vol) was added. Isolated yields.

**Table 3:** Results of quantum chemical calculations for studied compounds **2a**–**f**.

Compound	Total Energy, a.u.	E_(HOMO)_, eV	E_(LUMO)_, eV	∆E_(L-H)_, eV
**2a**	−1183.31	−5.789	−1.670	4.119
**2b**	−1298.58	−5.692	−1.551	4.142
**2c**	−3755.00	−5.751	−1.675	4.076
**2d**	−6326.70	−5.715	−1.645	4.071
**2e**	−3869.55	−5.564	−1.556	4.008
**2f**	−1104.56	−5.785	−1.653	4.132

**Table 4:** Frontier orbitals of several studied compounds.

Compound	HOMO	LUMO
**2a**	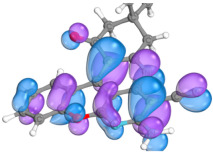	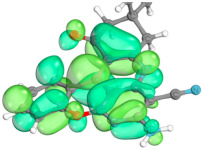
**2b**	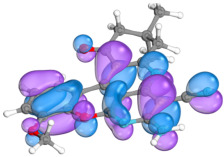	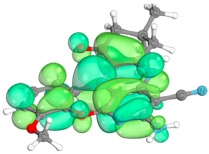
**2c**	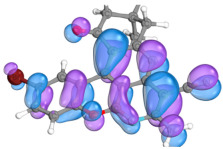	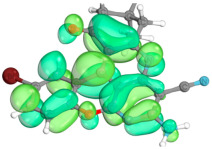
**2d**	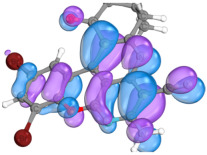	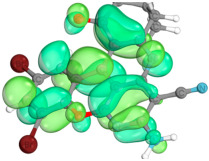
**2e**	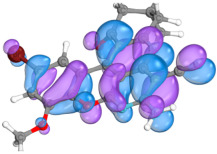	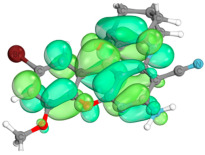
**2f**	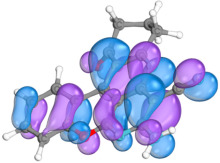	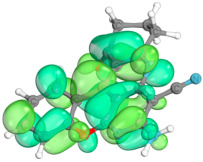

## Data Availability

Data is contained within the article or Supplementary Material.

## Supplementary Materials

The following supporting information can be downloaded at: https://www.mdpi.com/article/10.3390/molecules27134156/s1, Figures S1–S12: ^1^H and ^13^C Spectra of synthesized compounds **2a**–**f**, Figures S13–S17: 1D-NMR and 2D-NMR Spectra of **2a**, Figures S18 and S19: ^1^H-NMR monitoring spectra (300 MHz, 353 K).

## Sample Availability

Samples of the compounds **1a**–**f** and **2a**–**f** are available from the authors.
