# Supplementary material for: Oxidative Cyclization of 5H-Chromeno[2,3-b]pyridines to Benzo[b]chromeno[4,3,2-de][1,6]naphthyridines, Their NMR Study and Computer Evaluation as Material for LED
Source: Molecules. 2022 Jun 28;27(13):4156. doi: 10.3390/molecules27134156 (PMC9268656; doi:10.3390/molecules27134156)

Supplementary Material:

Oxidative Cyclization of 5H-Chromeno[2,3-*b*]pyridines to Benzo[b]chromeno[4,3,2-*de*][1,6]naphthyridines, their NMR Study and Computer Evaluation as Material for LED

Yuliya E. Ryzhkova, Fedor V. Ryzhkov, Artem N. Fakhrutdinov, Michail N. Elinson*

N. D. Zelinsky Institute of Organic Chemistry, Russian Academy of Sciences, 47 Leninsky Pr., Moscow 119991, Russian Federation. E-mail: elinson@ioc.ac.ru

Table of Contents

1. The copies of ^{1}H NMR and ^{13}C NMR spectra for compounds **2a-f**.. S2

2. 1D NMR and 2D NMR data (600 MHz) for compound **2a**.. S8

3. ^{1}H NMR monitoring spectra... S11

1. The copies of ^{1}H NMR and ^{13}C NMR spectra for compounds 2a-f

Figure S1. ^{1}H NMR spectrum of 7-amino-11,11-dimethyl-13-oxo-10,11,12,13-tetrahydrobenzo-[*b*]chromeno[4,3,2-*de*][1,6]naphthyridine-8-carbonitrile **2a** in DMSO-d_6.

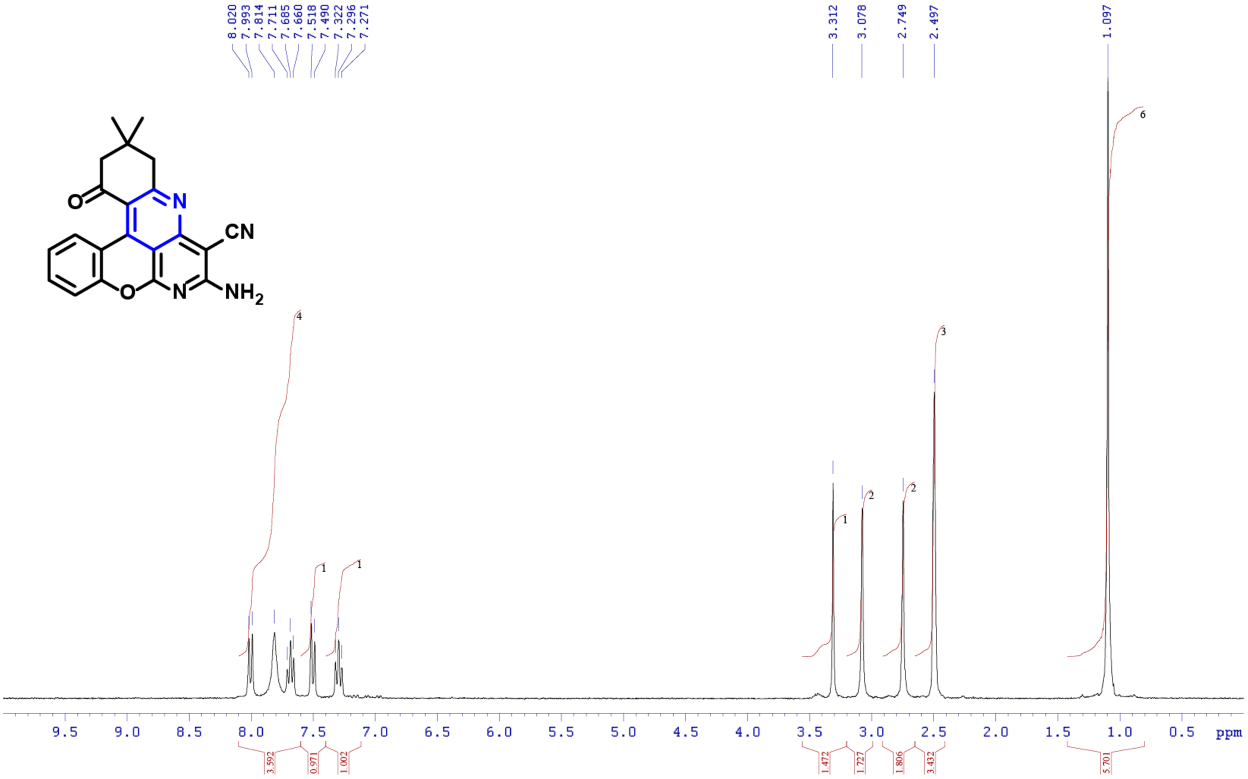

Figure S2. ^{13}C NMR spectrum of 7-amino-11,11-dimethyl-13-oxo-10,11,12,13-tetrahydrobenzo[*b*]chromeno[4,3,2-*de*][1,6]naphthyridine-8-carbonitrile **2a** in DMSO-d_6.

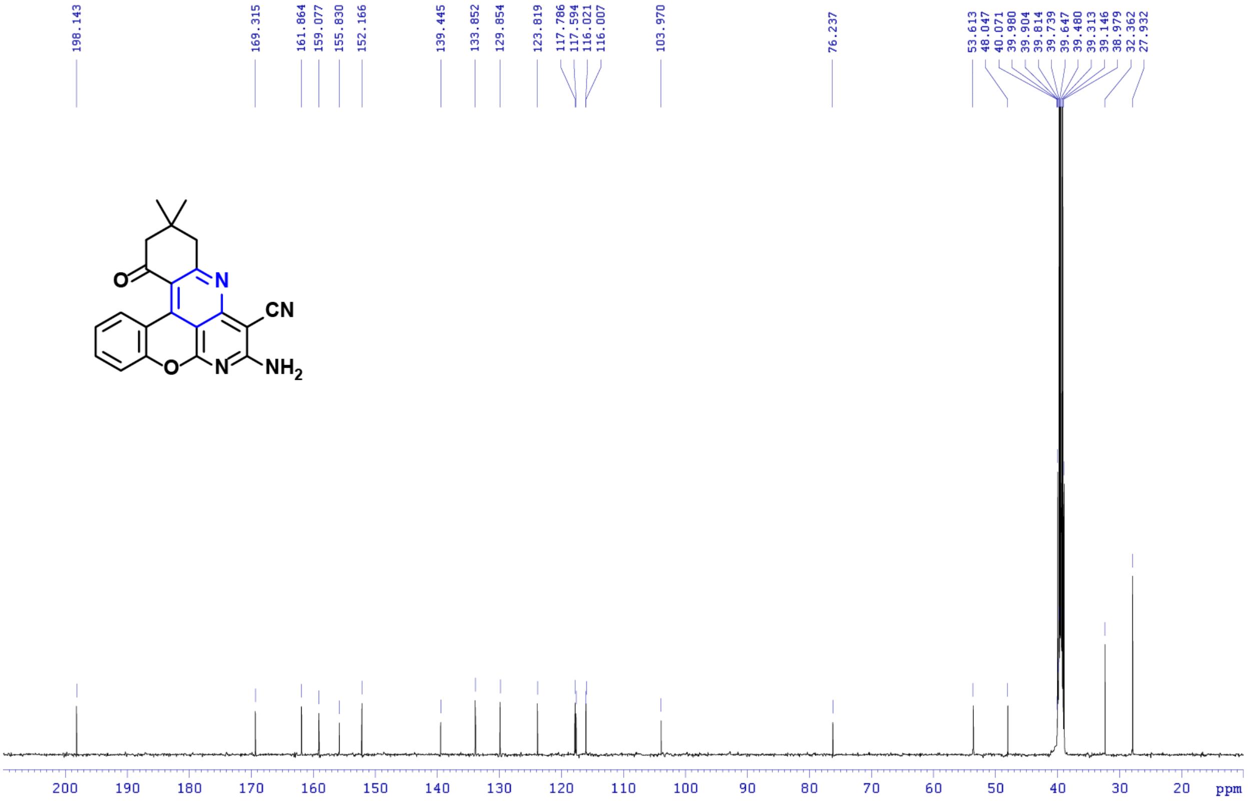

Figure S3. ^{1}H NMR spectrum of 7-amino-4-methoxy-11,11-dimethyl-13-oxo-10,11,12,13-tetrahydrobenzo[*b*]chromeno[4,3,2-*de*][1,6]naphthyridine-8-carbonitrile **2b** in DMSO-*d*$_6$.

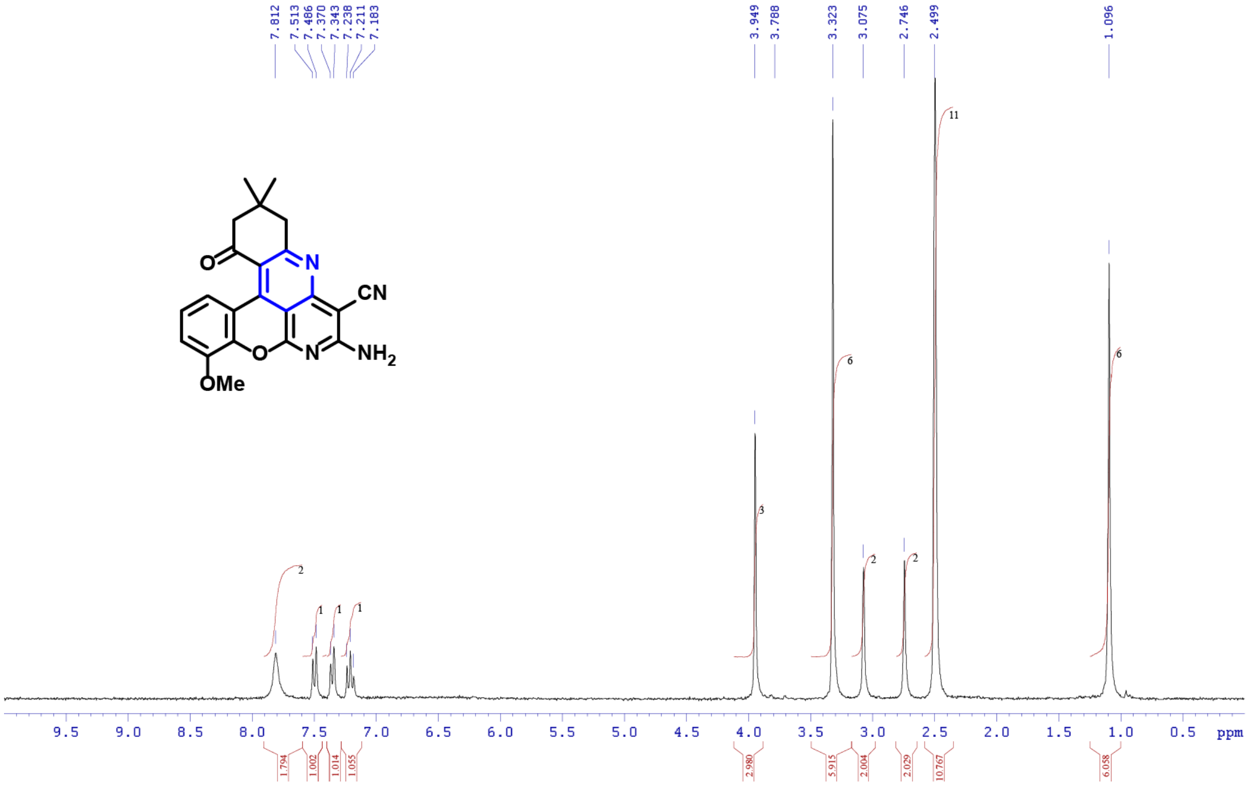

Figure S4. ^{13}C NMR spectrum of 7-amino-4-methoxy-11,11-dimethyl-13-oxo-10,11,12,13-tetrahydrobenzo[*b*]chromeno[4,3,2-*de*][1,6]naphthyridine-8-carbonitrile **2b** in DMSO-*d*$_6$.

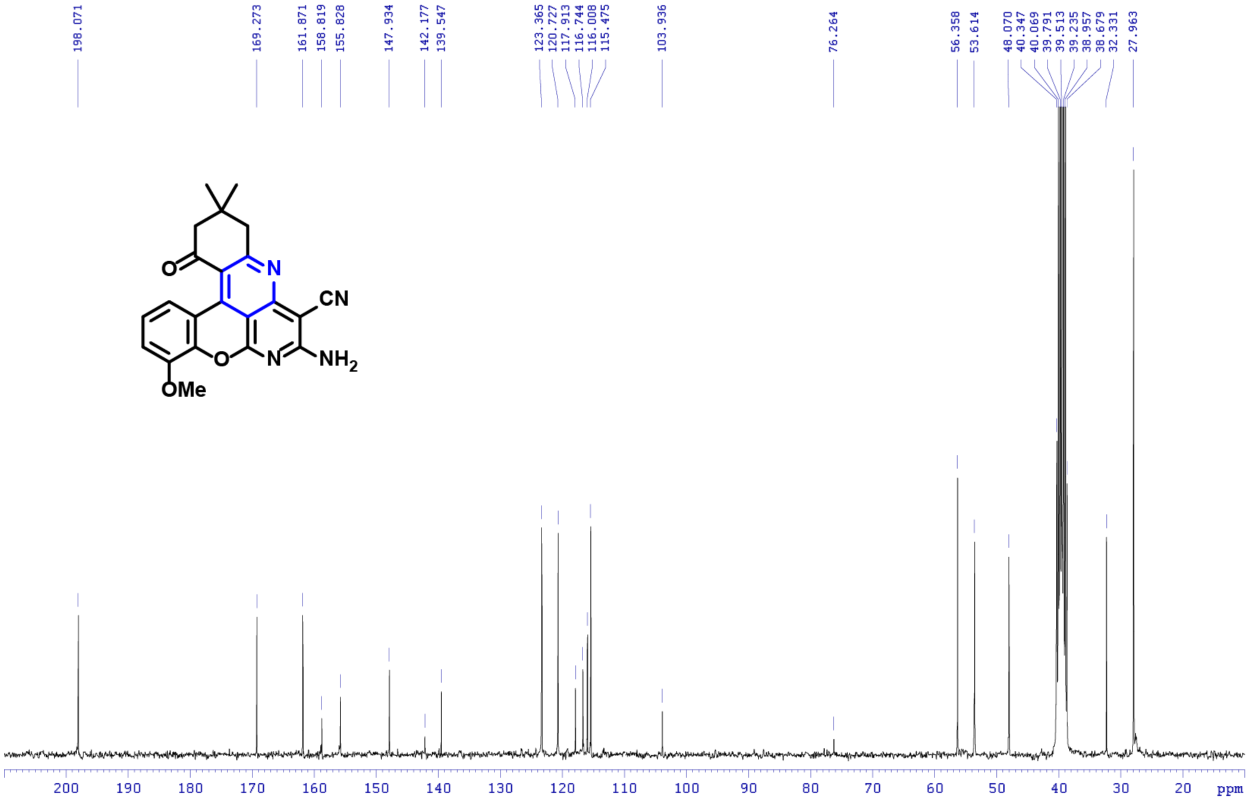

Figure S5. ^{1}H NMR spectrum of 7-amino-2-bromo-11,11-dimethyl-13-oxo-10,11,12,13-tetra-hydrobenzo[*b*]chromeno[4,3,2-*de*][1,6]naphthyridine-8-carbonitrile **2c** in DMSO-*d*$_6$.

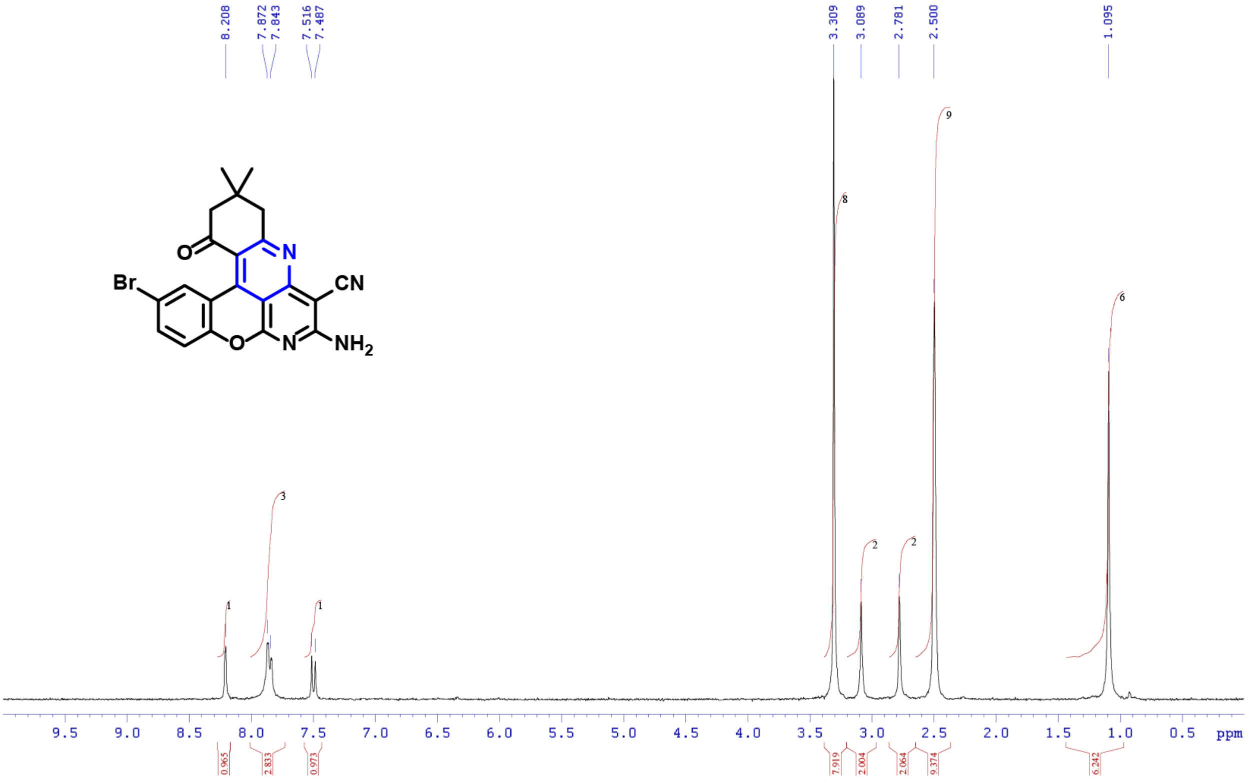

Figure S6. ^{13}C NMR spectrum of 7-amino-2-bromo-11,11-dimethyl-13-oxo-10,11,12,13-tetra-hydrobenzo[*b*]chromeno[4,3,2-*de*][1,6]naphthyridine-8-carbonitrile **2c** in DMSO-*d*$_6$.

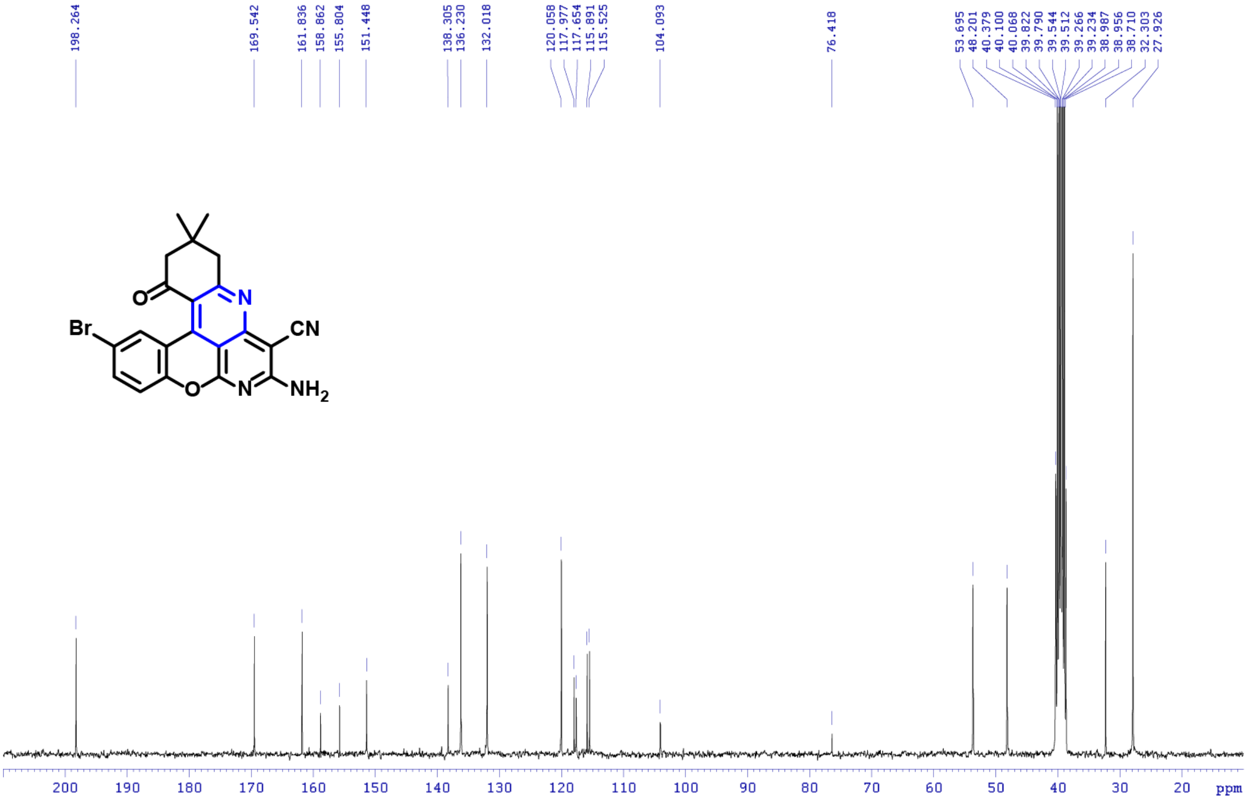

Figure S7. ^{1}H NMR spectrum of 7-amino-2,4-dibromo-11,11-dimethyl-13-oxo-10,11,12,13-tetrahydrobenzo[*b*]chromeno-[4,3,2-*de*][1,6]naphthyridine-8-carbonitrile **2d** in DMSO-d_6.

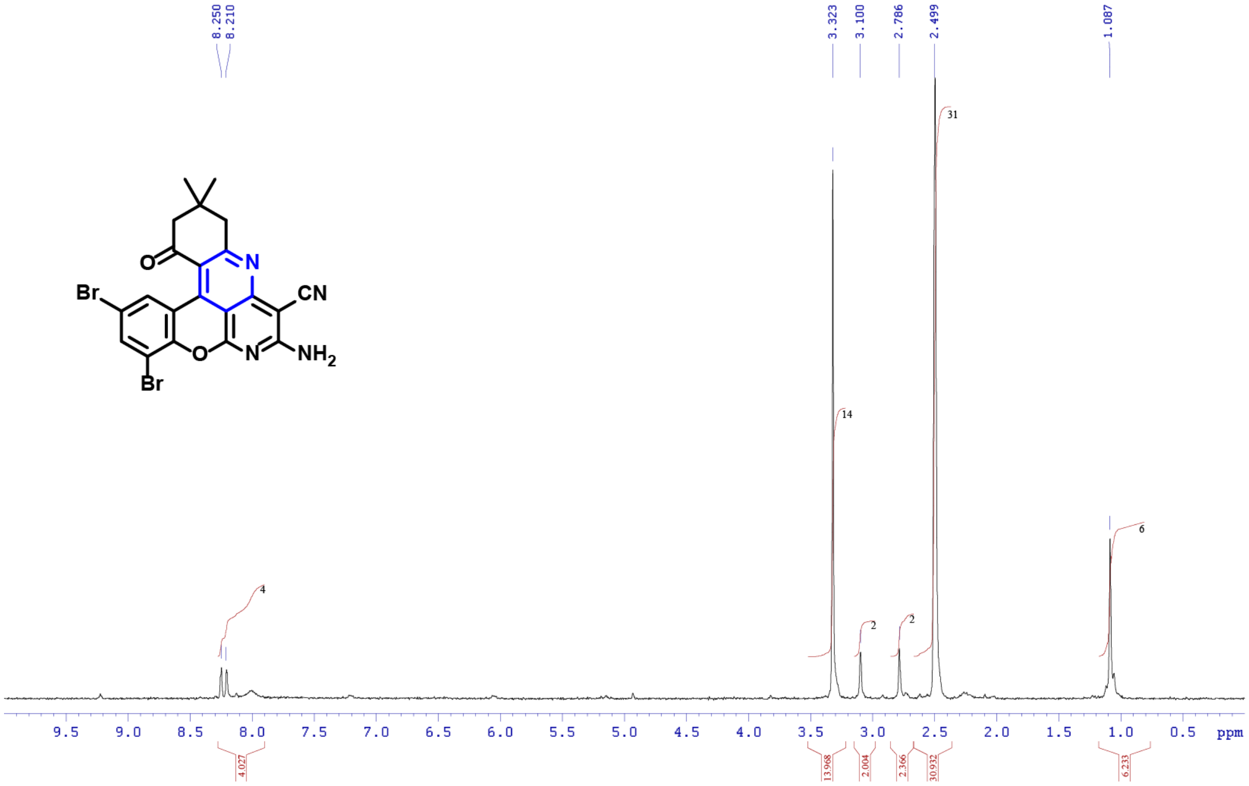

Figure S8. ^{13}C NMR spectrum of 7-amino-2,4-dibromo-11,11-dimethyl-13-oxo-10,11,12,13-tetrahydrobenzo[*b*]chromeno-[4,3,2-*de*][1,6]naphthyridine-8-carbonitrile **2d** in DMSO-d_6.

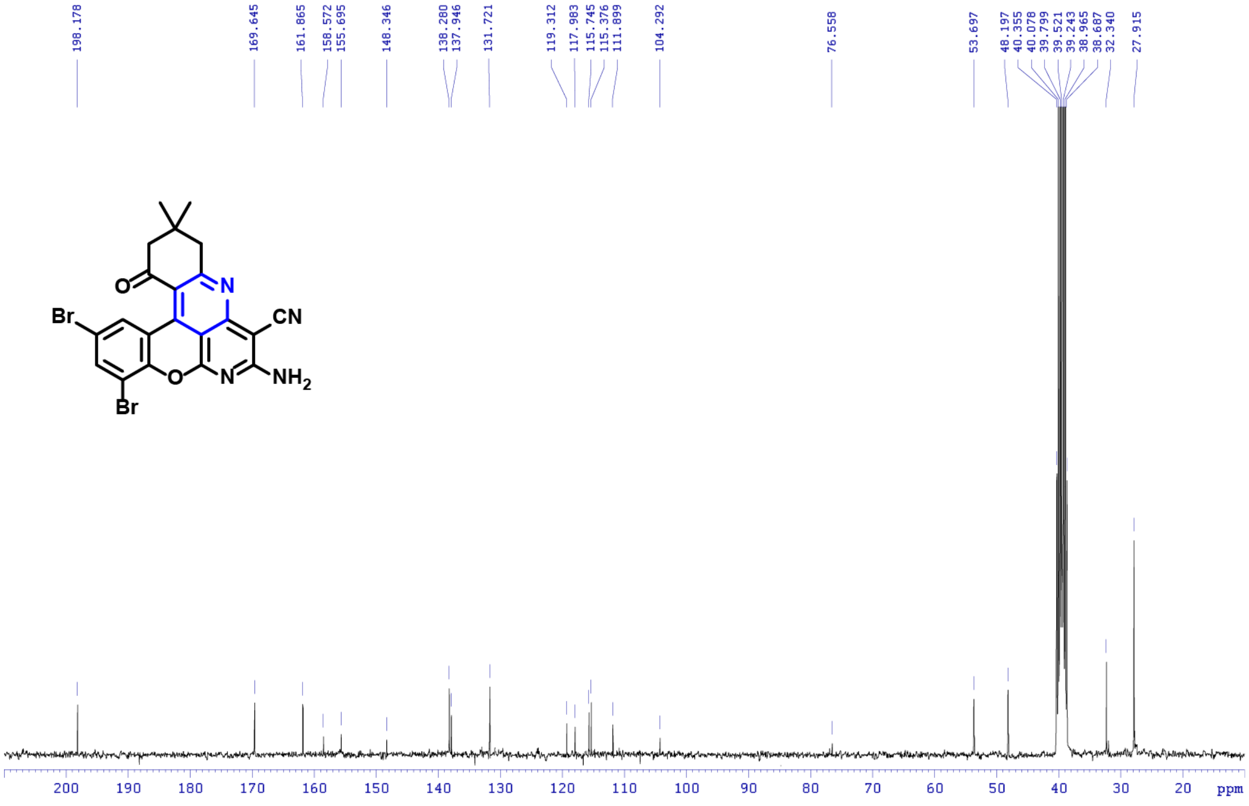

Figure S9. ^{1}H NMR spectrum of 7-amino-2-bromo-4-methoxy-11,11-dimethyl-13-oxo-10,11,12,13-tetrahydrobenzo[*b*]chromeno[4,3,2-*de*][1,6]naphthyridine-8-carbonitrile **2e** in DMSO-*d*$_6$.

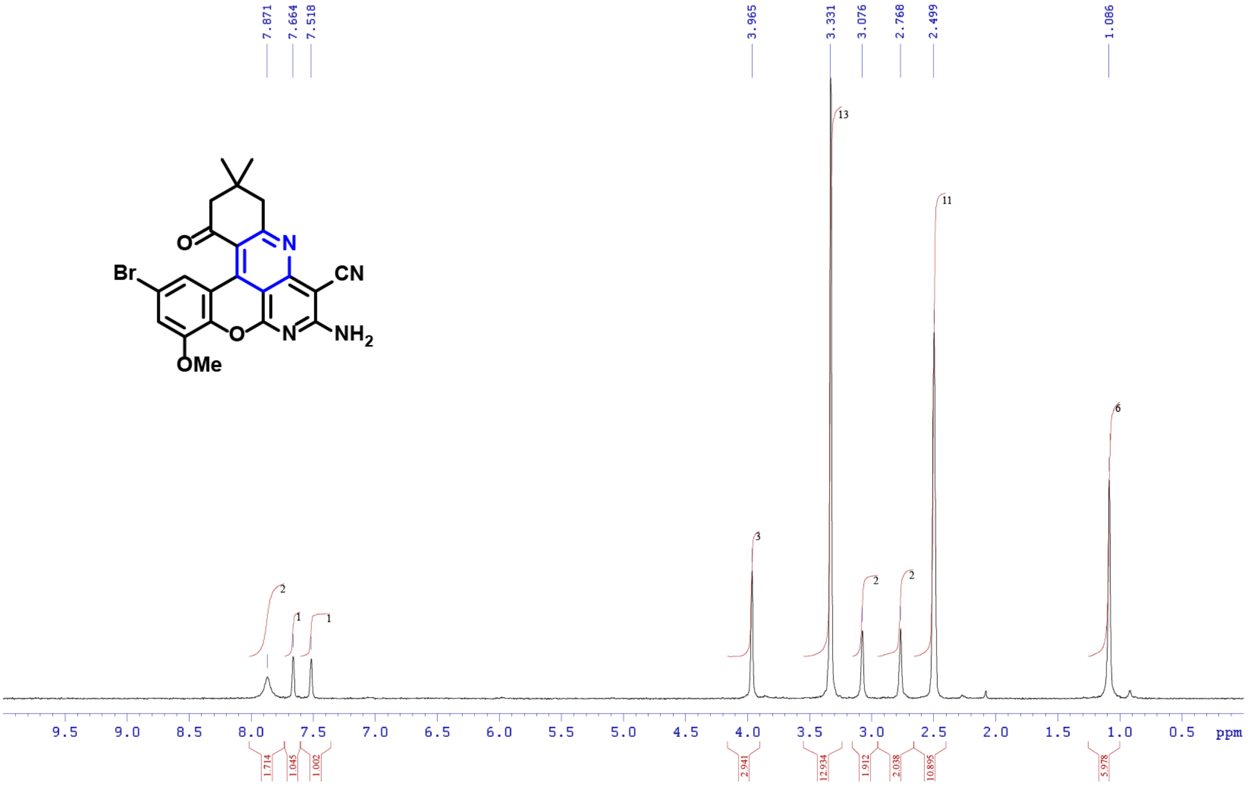

Figure S10. ^{13}C NMR spectrum of 7-amino-2-bromo-4-methoxy-11,11-dimethyl-13-oxo-10,11,12,13-tetrahydrobenzo[*b*]chromeno[4,3,2-*de*][1,6]naphthyridine-8-carbonitrile **2e** in DMSO-*d*$_6$.

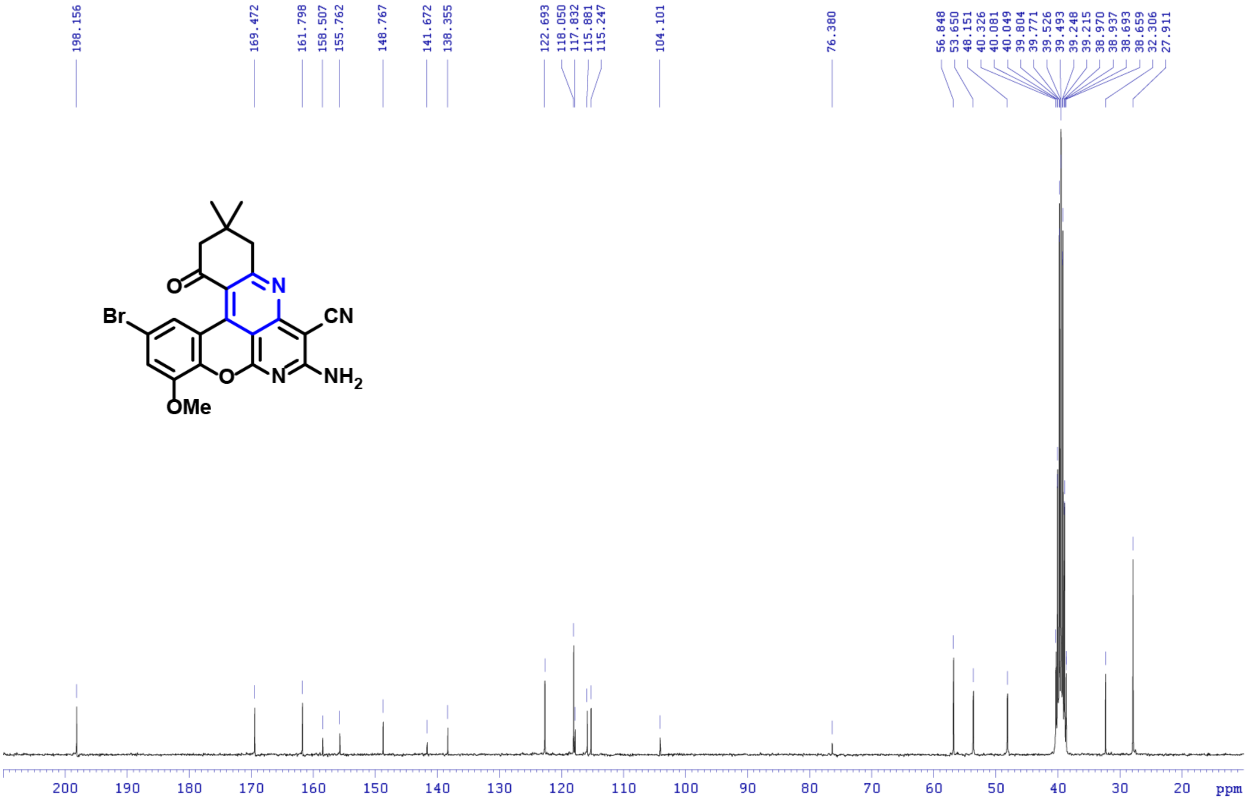

Figure S11. ^{1}H NMR spectrum of 7-amino-13-oxo-10,11,12,13-tetrahydrobenzo[*b*]-chromeno[4,3,2-*de*][1,6]naphthyridine-8-carbonitrile **2f** in DMSO-d_6.

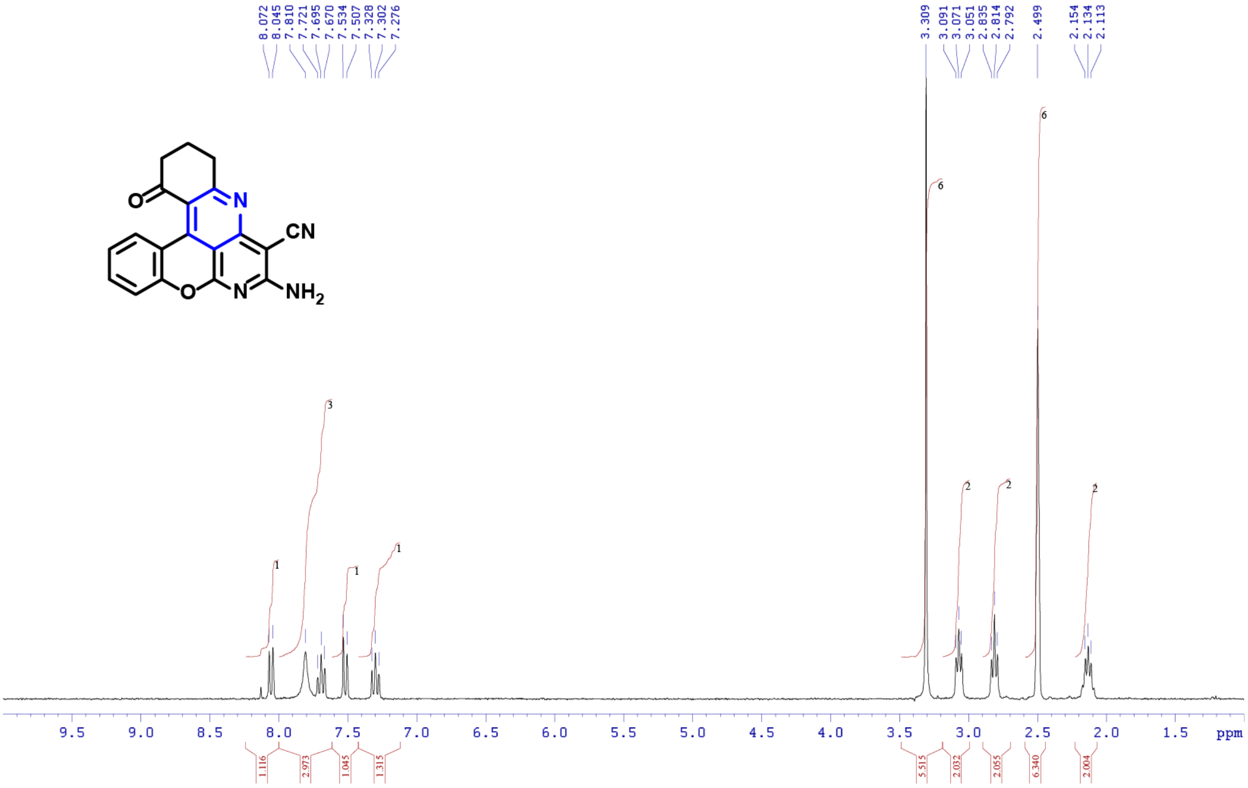

Figure S12. ^{13}C NMR spectrum of 7-amino-13-oxo-10,11,12,13-tetrahydrobenzo[*b*]-chromeno[4,3,2-*de*][1,6]naphthyridine-8-carbonitrile **2f** in DMSO-d_6.

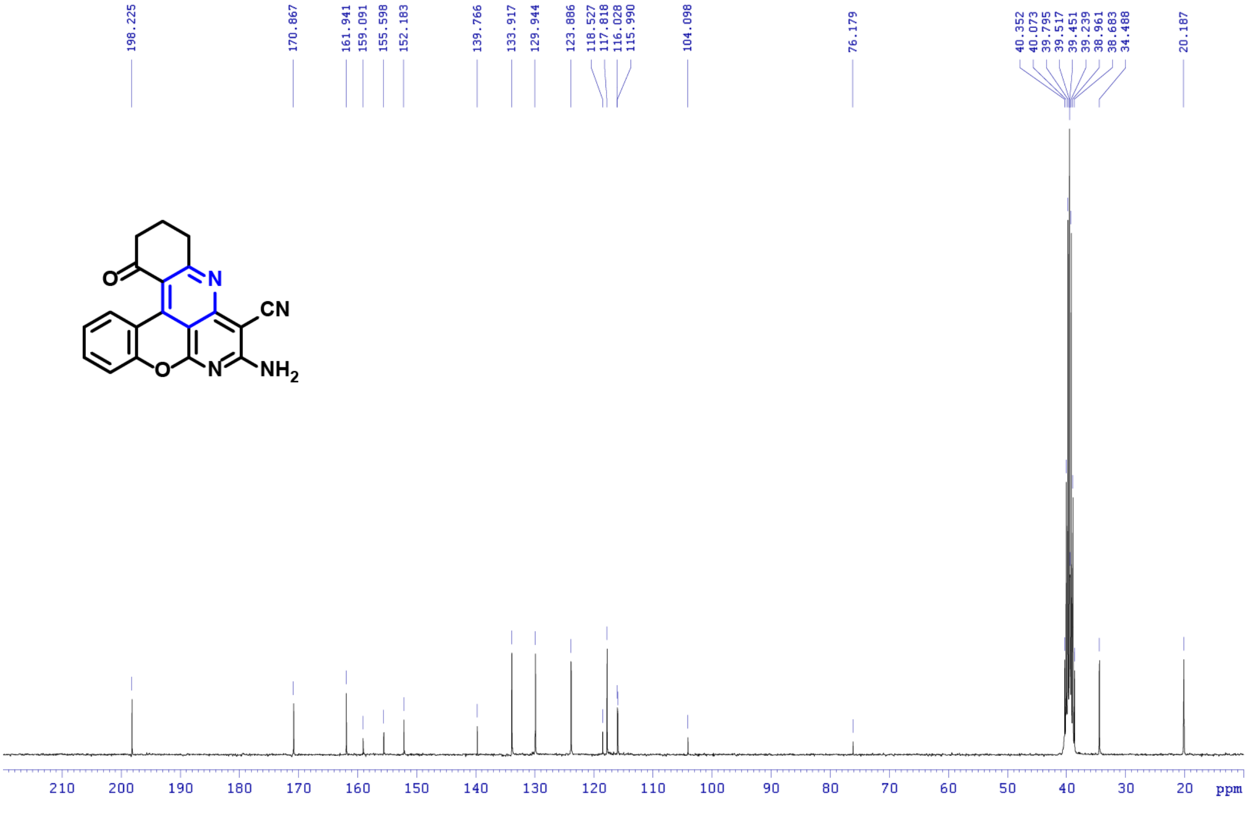

2. 1D NMR and 2D NMR data (600 MHz) for compound 2a

Figure S13. ^{1}H NMR spectrum of 7-amino-11,11-dimethyl-13-oxo-10,11,12,13-tetrahydrobenzo[*b*]chromeno[4,3,2-*de*][1,6]naphthyridine-8-carbonitrile **2a** in DMSO-*d*$_6$.

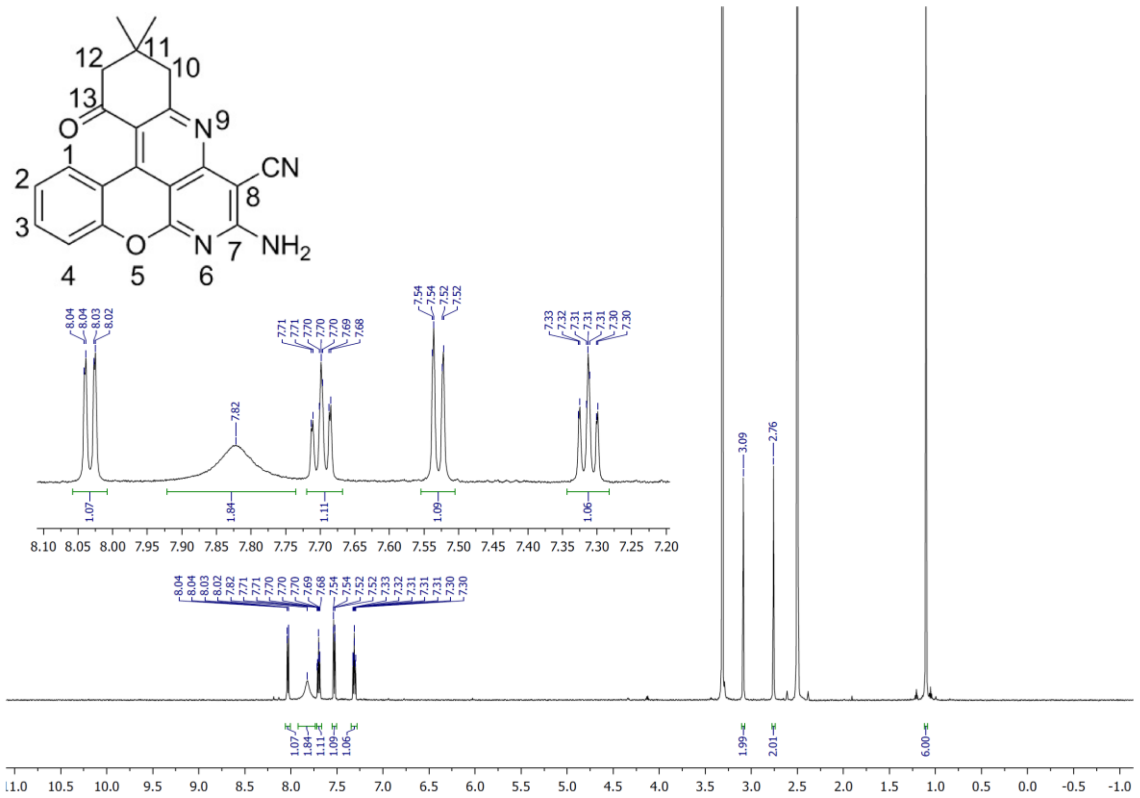

Figure S14. ^{13}C NMR spectrum of 7-amino-11,11-dimethyl-13-oxo-10,11,12,13-tetrahydrobenzo[*b*]chromeno[4,3,2-*de*][1,6]naphthyridine-8-carbonitrile **2a** in DMSO-*d*$_6$.

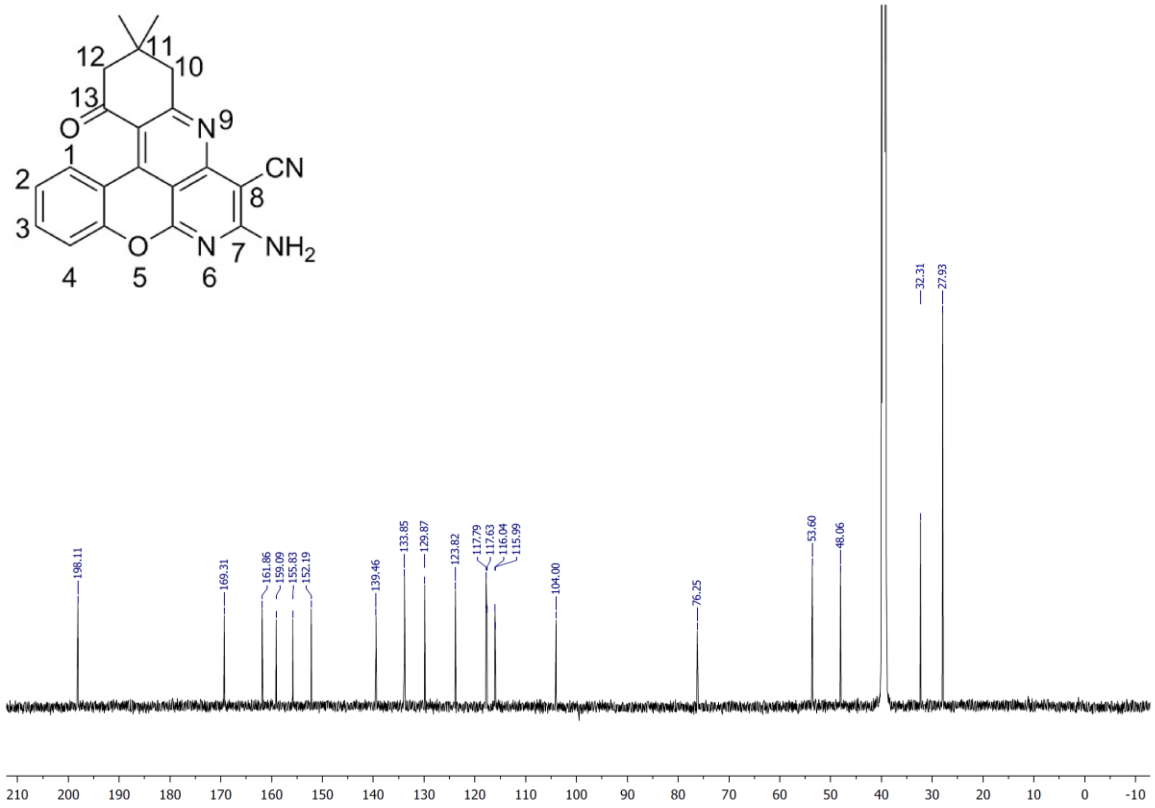

Figure S15. ^{1}H-^{1}H COSY NMR spectrum of 7-amino-11,11-dimethyl-13-oxo-10,11,12,13-tetrahydrobenzo[*b*]chromeno[4,3,2-*de*][1,6]naphthyridine-8-carbonitrile **2a** in DMSO-*d*$_6$.

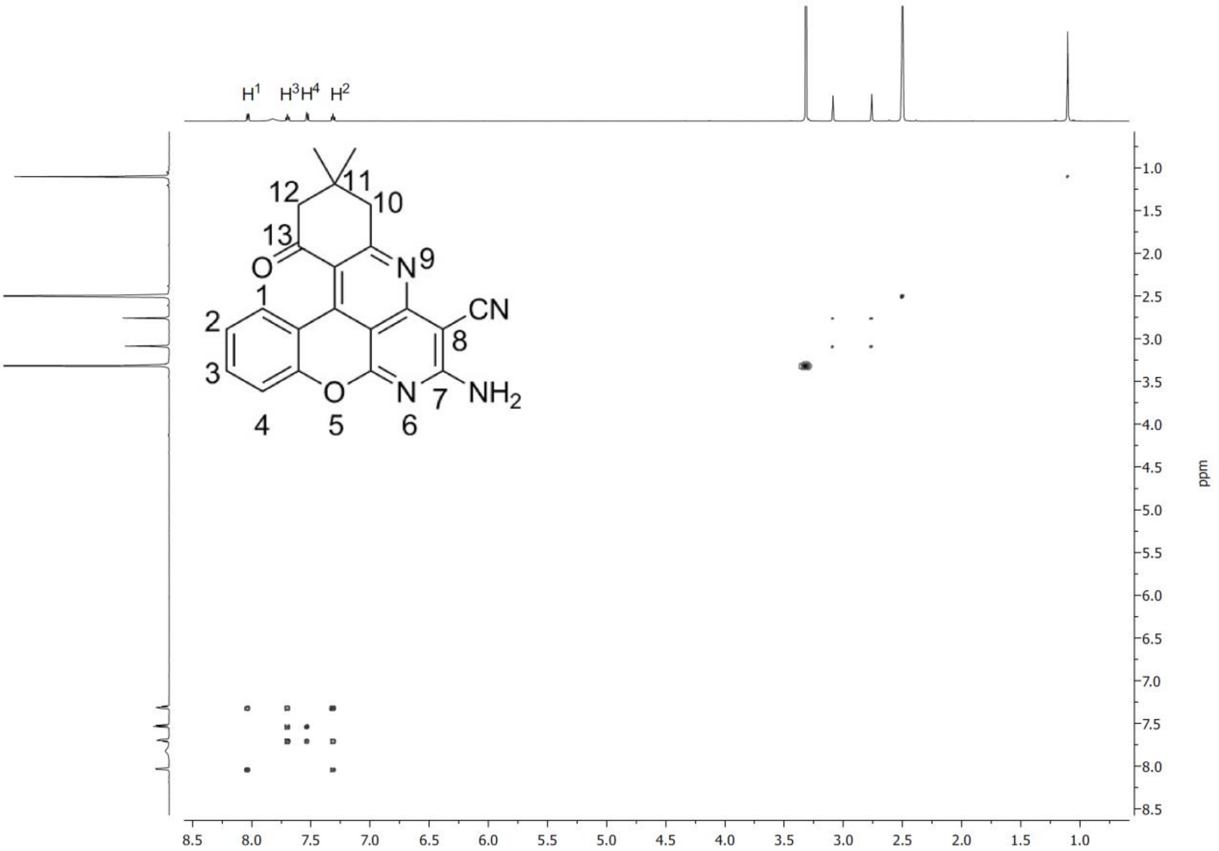

Figure S16. ^{1}H-^{13}C HSQC NMR spectrum of 7-amino-11,11-dimethyl-13-oxo-10,11,12,13-tetrahydrobenzo[*b*]chromeno[4,3,2-*de*][1,6]naphthyridine-8-carbonitrile **2a** in DMSO-*d*$_6$.

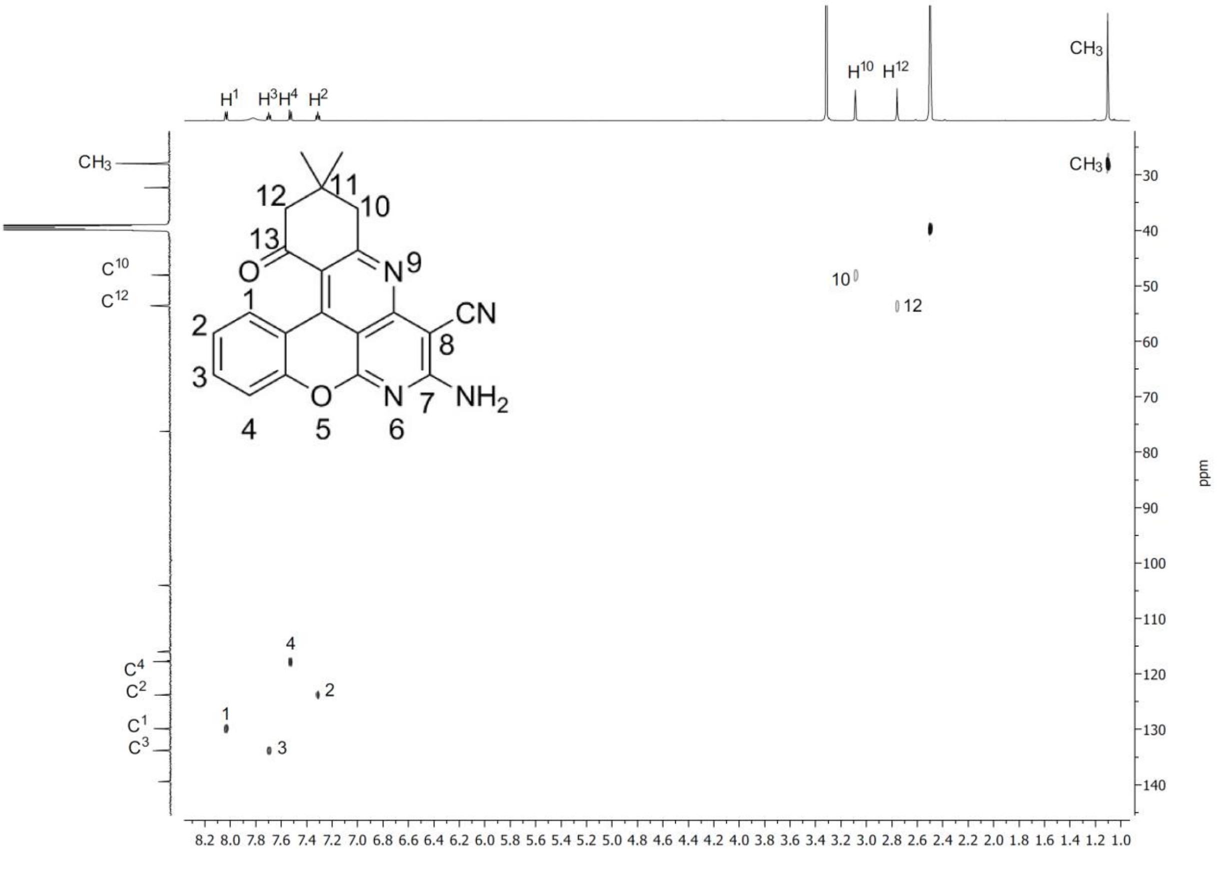

Figure S17. ^1H-^{13}C HMBC NMR spectrum of 7-amino-11,11-dimethyl-13-oxo-10,11,12,13-tetrahydrobenzo[*b*]chromeno[4,3,2-*de*][1,6]naphthyridine-8-carbonitrile **2a** in DMSO-d_6.

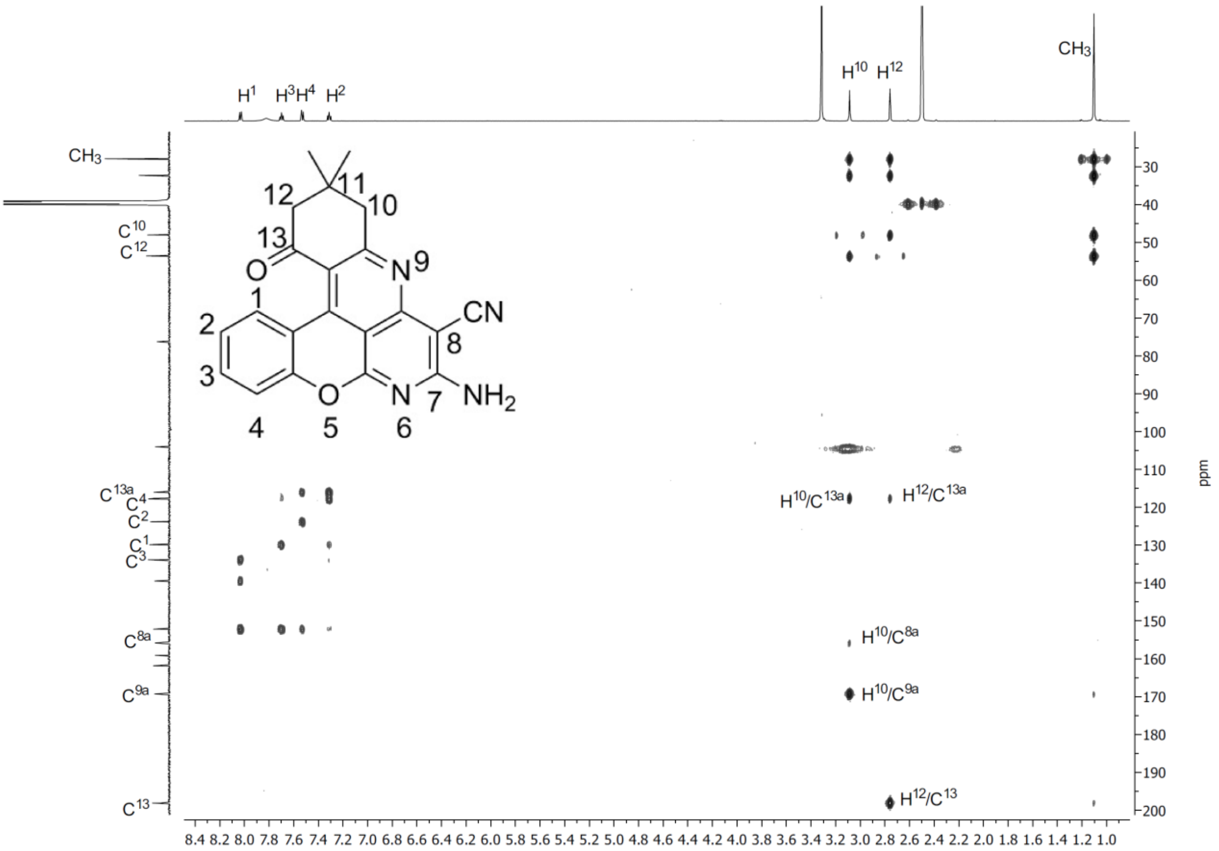

3. ^{1}H NMR monitoring spectra

Figure S18. Representative ^{1}H-NMR spectrum of intramolecular oxidative cyclization of chromeno[2,3-*b*]pyridine **1a** in DMSO-d_6 in the presence of formic acid at 353 K recorded after dissolution.

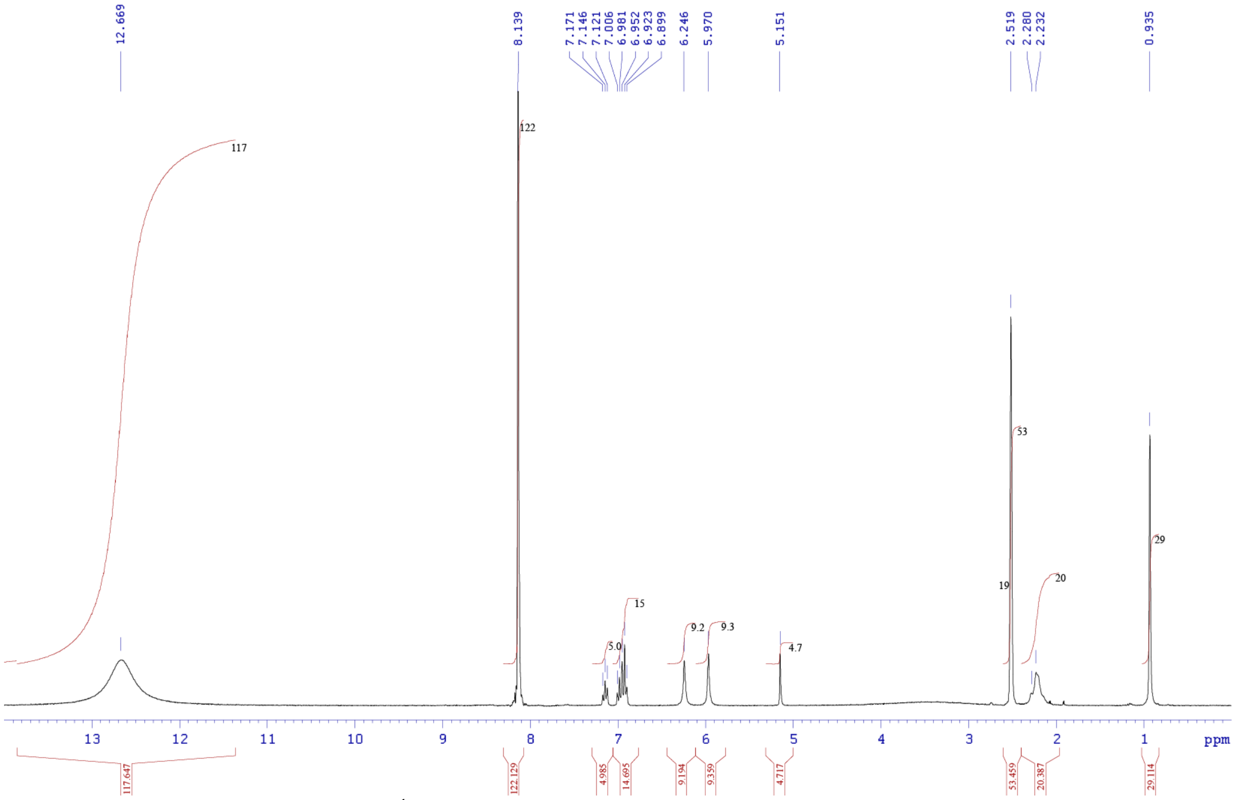

Figure S19. Representative ^{1}H-NMR spectrum of intramolecular oxidative cyclization of chromeno[2,3-*b*]pyridine **1a** in DMSO-d_6 in the presence of formic acid at 353 K recorded 60 min after dissolution.

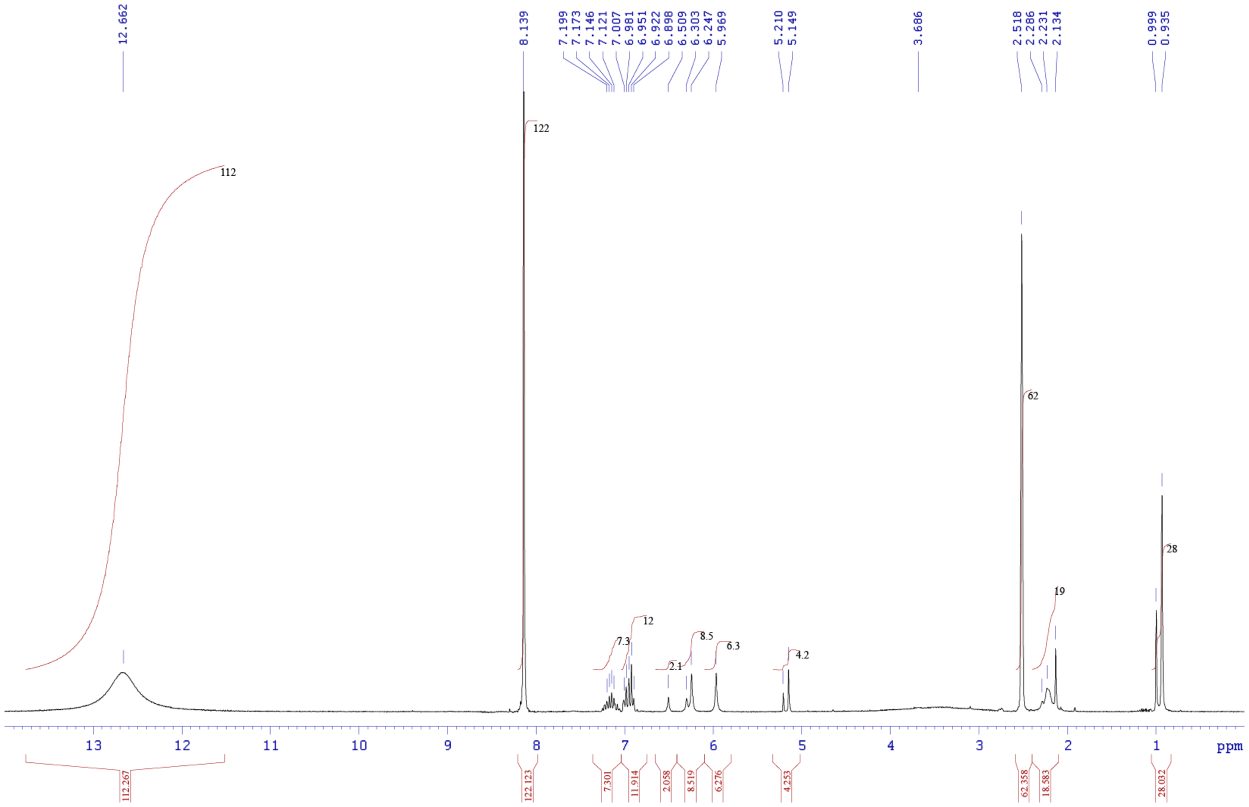

Supplement: Supplementary file 1 [file molecules-27-04156-s001.zip › molecules-1776279-supplementary.pdf]
